# Mutation detection in saliva from oral cancer patients

**DOI:** 10.1016/j.oraloncology.2024.106717

**Published:** 2024-04

**Authors:** Ahmed A. Ahmed, Mateja Sborchia, Hannah Bye, Maria Roman-Escorza, Ariella Amar, Rhonda Henley-Smith, Edward Odell, Mark McGurk, Michael Simpson, Tony Ng, Elinor J. Sawyer, Christopher G. Mathew

**Affiliations:** a School of Cancer and Pharmaceutical Sciences, Faculty of Life Sciences and Medicine, Guy’s Cancer Centre, King’s College London, London SE1 9RT, United Kingdom; b Department of Medical and Molecular Genetics, Faculty of Life Sciences and Medicine, King’s College London, London SE1 9RT, United Kingdom; c KHP Head & Neck Cancer Biobank, Guy’s & St Thomas’ NHS Foundation Trust, Guy’s Hospital, London SE1 9RT, United Kingdom; d King's College London and Head and Neck Pathology Guy's Hospital, London SE1 9RT, United Kingdom; e Department of Head and Neck Surgery, University College London Hospital, London NW1 2BU, United Kingdom; f Richard Dimbleby Laboratory of Cancer Research, School of Cancer and Pharmaceutical Sciences, King's College London, Guy's Medical School Campus, London SE1 1UL, United Kingdom; g Sydney Brenner Institute for Molecular Bioscience, University of the Witwatersrand, Johannesburg, South Africa

**Keywords:** Head and neck squamous cell carcinoma HNSCC, Oral squamous cell carcinoma OSCC, Saliva, Cell-free DNA, Circulating tumor DNA, Early detection

## Abstract

**Objectives:**

The incidence of head and neck squamous cell carcinoma (HNSCC) continues to increase and although advances have been made in treatment, it still has a poor overall survival with local relapse being common. Conventional imaging methods are not efficient at detecting recurrence at an early stage when still potentially curable. The aim of this study was to test the feasibility of using saliva to detect the presence of oral squamous cell carcinoma (OSCC) and to provide additional evidence for the potential of this approach.

**Materials and Methods:**

Fresh tumor, whole blood and saliva were collected from patients with OSCC before treatment. Whole exome sequencing (WES) or gene panel sequencing of tumor DNA was performed to identify somatic mutations in tumors and to select genes for performing gene panel sequencing on saliva samples.

**Results:**

The most commonly mutated genes identified in primary tumors by DNA sequencing were *TP53* and *FAT1*. Gene panel sequencing of paired saliva samples detected tumor derived mutations in 9 of 11 (82%) patients. The mean variant allele frequency for the mutations detected in saliva was 0.025 (range 0.004 – 0.061).

**Conclusion:**

Somatic tumor mutations can be detected in saliva with high frequency in OSCC irrespective of site or stage of disease using a limited panel of genes. This work provides additional evidence for the suitability of using saliva as liquid biopsy in OSCC and has the potential to improve early detection of recurrence in OSCC. Trials are currently underway comparing this approach to standard imaging techniques.

## Highlights

• Frequently mutated genes in OSCC: *TP53* (64%), *FAT1* (27%), *CDKN2A*, *CASP8* and *DNAH7.*
• Tumor somatic mutations of OSCC can be detected in saliva DNA at high sensitivity.
• The mutation detection is independent of primary site of the tumor or tumor stage.

## Introduction

Head and neck squamous cell carcinoma (HNSCC) is the eighth most common form of cancer in the United Kingdom and the sixth worldwide [1], [2]. HNSCC incidence rates have increased by 34 % in the UK between 1993 and 2018 and are expected to continue increasing worldwide [1], [2], [3].

HNSCCs are mainly derived from the mucosal epithelium in the pharynx, larynx and oral cavity and are divided into two types: 1) HPV-positive cancers, which are often oropharyngeal cancers; 2) HPV-negative cancers, which are mainly oral cavity and laryngeal cancers [3] and are associated with smoking and alcohol consumption. The HPV-negative HNSCCs tend to present at an advanced stage and have worse outcome compared to HPV-positive HNSCCs with survival rates of 11 % at 5 years [4]. Treatment depends on the stage of the cancer and generally involves a combination of surgery, radiotherapy and chemotherapy [3].

Many patients (31.2–62.6 %) develop locally recurrent disease [5], which if detected early can in some cases be treated successfully with salvage surgery. However, these recurrences are often difficult to detect on routine imaging due to the fibrosis caused by surgery and radiotherapy. It is, therefore, important to be able to detect recurrence or minimal residual disease following radical treatment early so that salvage surgery can be performed while the disease is still resectable.

Cell-free tumor DNA (cfDNA) in plasma may provide a more sensitive method to detect early recurrence or minimal residual disease in many different cancer types, with studies showing detection 3.5–4 months before standard imaging in melanoma and HNSCC [6], [7]. Cell-free tumor DNA (cfDNA) can also be detected in saliva, urine and cerebrospinal fluid [8], [9]. The ideal source of cfDNA depends on the cancer type. For instance, cerebrospinal fluid is a better source than plasma for brain tumors [9]. It therefore follows that saliva may be an important source of cfDNA in HNSCC and there is some early evidence that this is the case in oral cancers [10]. In this study, we aim to provide additional evidence for the feasibility of using saliva to detect somatic tumor mutations in oral squamous cell carcinoma (OSCC).

## Materials and methods

### Patient samples

14 patients diagnosed with OSCC between 2013 and 2014 at Guys and St Thomas Hospital Foundation Trust were included in this study with ethical approval (REC 14/LO/0300) and informed consent. The median age was 59.5 years, ranging from 41 to 71 years (5 female, 9 male). Tumor, whole blood and saliva were collected from each patient before treatment and stored for subsequent analysis. The clinic-pathological features are summarized in Table 1. Saliva was collected using the ORAgene DNA saliva collection kit (DNAGenotek Inc, Ontario).

**Table 1:** Summary of the clinical data of patients used in the study.

**Patient No**	**Age at diagnosis**	**Sex**	**Tumor site**	**TNM stage**	**Differentiation**
1	71	F	Right lower alveolus	T4aN2cM0	Moderate
2	84	F	Left upper alveolus	T4aNxM0	Moderate
3	55	F	Right buccal mucosa	T4aN0M0	Poor
4	67	F	Left buccal mucosa	T2N1M0	Poor
5	71	F	Right lower alveolus	T4N2bM0	Poor
6	48	M	Right lower alveolus	T4aN2bM0	Moderate
7	67	M	buccal mucosa/lip	T2N0	Poor
8	54	M	Left lower alveolus	T4aN1M0	Poor
9	41	M	Right retromolar trigone	T3N0M0	Moderate
10	48	M	Floor of mouth	T4aN0	Moderate
11	62	M	Left tongue	T4aN2cM0	Moderate
12	53	M	Left tongue	T2N2bM0	Moderate
13	64	M	Midline floor of mouth	T4N0M0	Moderate
14	57	M	Right buccal mucosa	T3N0M0	Well

### DNA extraction

Tumor tissue was microdissected from fresh frozen sections. Tumor and matched blood DNA were extracted using the QIAamp DNA Mini Kit (Qiagen). Saliva was collected using the ORAgene kit and DNA was extracted using the prepIT.L2P extraction kit (DNAGenotek Inc, Ontario) according to the manufacturer’s instructions. The required amount of DNA (10–100 ng) was cleaned prior to library preparation for all saliva samples using AMPure® XP Beads (Beckman Coulter, Inc). DNA concentration was quantified by Qubit 2.0 fluorometer using Quant-iT™ PicoGreen® or Qubit dsDNA BR Assay kits (ThermoFisher).

### Whole exome sequencing of blood and tumor DNA

Tumor and paired germline DNA (extracted from whole blood) were used to prepare WES libraries using the SureSelect Human All Exon V4 kit (Agilent) as per the manufacturer’s instructions. Libraries were sequenced on an Illumina HiSeq 2000 to a coverage of 100x in the Guy’s and St Thomas’ NHS Foundation Trust Biomedical Research Centre’s Genomics Facility. The sequencing reads were trimmed at the 5′ end to remove primer sites using Btrim and the trimmed reads were then aligned to the reference human genome hg19 using NovoAlign (https://www.novocraft.com/products/novoalign/). Duplicated reads were marked by Picard (v1.112) [11] and aligned marked BAM files were indexed by Samtools (v0.1.19) [12]. The BAM files were then analyzed by MuTect (v1.1.4) [13] and Pindel (v0.2.5b9) [14] against the matched normal sample to detect somatic single nucleotide variants (SNV) and insertions/deletions (indels), respectively.

Variants were only considered if they passed the following quality control (QC) criteria: exonic variants with tumor coverage ≥ 15x and a tumor allele fraction ≥ 0.05, with normal sample coverage ≥ 10x and normal allele fraction < 0.05. Variants with a population frequency > 0.01 in ExAC, ESP6500, or 1000 g databases were excluded. An additional filter was applied to missense variants in order to identify the most likely driver mutations by considering only missense variants reported in the COSMIC database. The variant allele frequency (VAF) was calculated by dividing the number of mutant reads by the total number of reads at each position of the mutant nucleotide.

For three tumor samples for which there was no paired germline DNA for WES, somatic tumor mutations were identified using the same Panelseq protocol described below for cell-free DNA. 100 ng of tumor DNA was used in library preparation Illumina kit including a fragmentation step (Nonacus Ltd.). In order to remove any potential germline mutations, only exonic mutations reported in COSMIC database with an allele fraction in the tumor sample ≥ 0.01 and with a population frequency < 0.01 in ExAC [15], ESP6500 [16], and 1000 g [17] databases were retained.

### Panelseq of saliva DNA

A custom panel of 12 genes was designed using the Nonacus platform (Nonacus Ltd.) for use in this OSCC project and a separate breast cancer project. The selection of genes for OSCC was based on the genes found to be mutated in the initial analysis of WES data from the tumors in our study and included the following genes: *TP53*, *CDKN2A*, *KDM6B*, *NSD1*, *DNAH7, PIK3CA* for OSCC and *PTEN*, *FOXA1*, *CDH1*, *TBX3*, *RUNX1*, *RBL1* for breast cancer.

Between 10 and 100 ng of fragmented saliva DNA was used in library preparation using the Cell3 Target Cell-Free DNA Target Enrichment Illumina kit with the custom gene panel (Nonacus Ltd.) as per the manufacturer’s instructions. All samples were PCR enriched with 5–9 PCR cycles depending on the amount of DNA used. QC of DNA libraries was performed using an Agilent 4200 TapeStation to check the quality and peak size of the library and to check that neither primer dimer nor other unexpected peaks were present. The libraries’ concentration was measured with a Qubit 2.0 fluorometer. Paired-end 100 bp sequencing of libraries was done on a NextSeq 2000 sequencer (Illumina) to a mean depth of 20,000x. FASTQ files were aligned to the human reference genome GRCh38 (hg38). Removal of unique molecular identifiers (UMIs) and consensus BAM file preparation were performed using NonacusTools (v1.0). BAM files were then marked for duplications and variants were called using MuTect2 from the Genome Analysis Toolkit (v.4.1.0.0). The called mutations in saliva samples were then compared to the somatic mutations in tumors to confirm the presence of shared mutations. Mutations were visualized on the Integrative Genomics Viewer (IGV) to confirm the presence of the shared mutations. For any potential somatic mutations detected only in saliva, the same genomic region in the tumor was visualized on the IGV to confirm that the mutation was not present in the tumor. In two cases, frameshift deletions in *TP53* (Patients 2 and 13) were identified in the tumor that had been filtered out by QC criteria. These mutations were reinstated into the list of tumor somatic mutations.

### Statistical analysis

All statistical analyses were done using Prism e.g. two-sided Student’s *t*-test.

## Results

### Clinical features of HNSCC patients

A total of 14 patients were recruited for this feasibility study. All patients were diagnosed with OSCC between 2013–2014. The median age was 59.5 years (range 41 – 71). There were 9 males (64 %) and 5 females (36 %) with females being older than males by an average of 15 years (P = 0.02, Student’s *t*-test). Most patients had locally advanced OSCC (nine T4 tumors and two T3 tumors). Although all patients had OSCC, the exact site of the primary differed between patients and included lower alveolus (29 %), buccal mucosa (29 %), tongue (14 %), floor of mouth (14 %), upper alveolus (7 %) and retromolar trigone (7 %). The clinical details of the patients are shown in Table 1. No details were available on HPV status as these samples were collected before routine testing was implemented in the UK.

### Mutations in primary tumors

**Figure 1:**
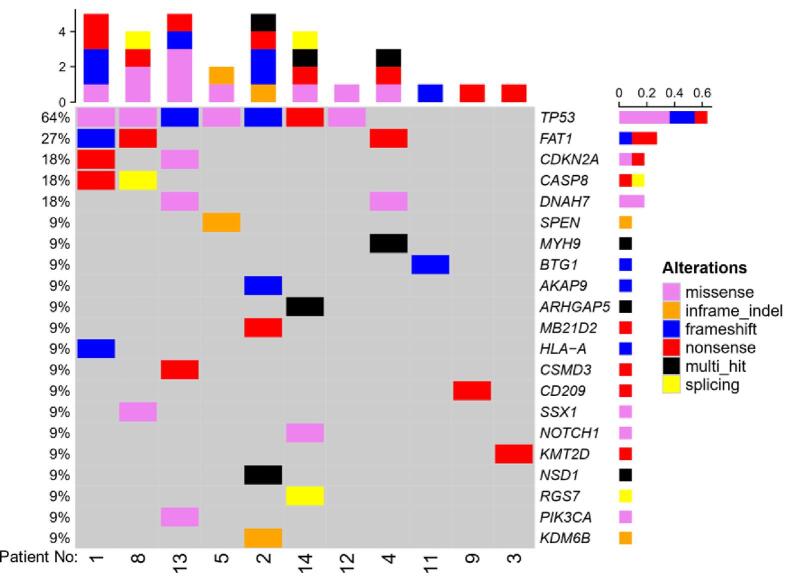
OncoPlot of 11 OSCC tumor samples showing mutated genes derived from WES data.

WES was performed on 11 patient tumor samples and matched germline DNA from whole blood. The most frequently mutated genes were *TP53* (64 %), *FAT1* (27 %), and *CDKN2A*, *CASP8* and *DNAH7* (18 % each) (Figure 1). The most prominent mutation types were missense mutations and premature stop codons (nonsense mutations). The remaining three patient tumors did not have paired germline DNA available and underwent Panelseq using the Nonacus custom platform. Somatic mutations were identified in all three and included two well-characterized mutations in *TP53* (p.Q65X) and (p.V157F), and a truncating *PTEN* mutation (Q171X). Overall, 9 of the 14 tumors (64 %) had a mutation in *TP53*.

### Detection of tumor mutations in saliva

Three of 14 patients were not included in the downstream analysis of saliva DNA. In two patients (Patients 3 and 11), the somatic mutations in these tumors (in *KMT2D* and *BTG1*) were detected in a re-analysis of the WES data and these two genes were therefore not included in the original Panelseq design. Insufficient saliva DNA was available from the third patient (Patient 14) for sequencing.

In the nine of 11 samples where somatic driver mutations were detected in the tumor, we were able to detect the identical somatic mutations in the saliva (Table 2). The mean of variant allele frequency (VAF) for the mutations detected in saliva was 0.025 (range 0.004 – 0.061). Detection rates of mutations in saliva samples of OSCC patients for this and other similar studies are summarized in Table 3.

**Table 2:** Summary of shared somatic mutations between tumor and saliva DNA.

**Patient No**	**TNM**	**Tumor Method**	**Mutation in tumor**	**VAF tumor**	**Found in saliva**	**VAF saliva (Mutant/ Total Reads)**
1	T4aN2cM0	WES	CDKN2A: p.R80X	0.476	Yes	0.032 (3/94)
			TP53: p.R248W	0.513	Yes	0.013 (3/236)
2	T4aNxM0	WES	KDM6B: p.263_264del	0.158	Yes	0.057 (24/418)
			TP53: p.G108Vfs*14	0.667	Yes	0.018 (10/546)
			NSD1: p.S1229L	0.202	Yes	0.004 (2/546)
5	T4N2bM0	WES	TP53: p.R282W	0.382	Yes	0.005 (3/550)
9	T3N0M0	WES	TP53: c.782 + 1G > A	0.636	Yes	0.038 (15/397)
12	T2N2bM0	WES	TP53: p.Y205C	0.622	Yes	0.008 (10/1324)
13	T4N0M0	WES	CDKN2A: p.M53I	0.300	Yes	0.022 (14/630)
			TP53: p.A86Vfs*54	0.083	Yes	0.015 (13/861)
			PIK3CA: p.K548N	0.184	Yes	0.058 (16/275)
4	T2N1M0	WES	DNAH7: p.S8L	0.192	No^#^	<0.004 (1/252)
8	T4aN1M0	WES	TP53: p.R273H	0.134	No	<0.002 (0/574)
6	T4aN2bM0	Panelseq	TP53: p.V157F	0.950	Yes	0.034 (71/2098)
7	T2N0	Panelseq	PTEN: p.Q171X	0.543	Yes	0.008 (7/864)
10	T4aN0	Panelseq	TP53: p.Q65X	0.016	Yes	0.007 (12/1696)

^#^The one mutant read in this saliva sample did not pass calling criteria.

**Table 3:** Comparison of published detection rates using saliva in OSCC.

Study	Saliva Component	Mutation method	Detection % in saliva	Ref
This Study	Whole DNA	12 gene Panelseq	82	
Wang et al.	Whole DNA	ddPCR	100	[10]
Cui et al.	cfDNA only	71 gene Panelseq	91	[7]
Shanmugam et al.	Cellular DNA only	7 gene-Panelseq	93	[31]

For the two samples in which we could not detect the somatic mutation in the saliva (Patients 4 and 8), their somatic mutations had a tumor VAF < 20 %. Interestingly, tumor mutations were detectable in saliva in all stages of the disease and there was no association with the anatomical site of the tumor.

## Discussion

This study investigated the utility of saliva DNA for the detection of somatic mutations in patients with OSCC. All tumor samples were initially sequenced to identify somatic mutations prior to detection in the saliva. The genes most frequently mutated in the primary tumors were *TP53* (64 %), *FAT1* (27 %), *CDKN2A* (18 %), *CASP8* (18 %) and *DNAH7* (18 %). Although our sample size was small, these findings are consistent with larger studies of HNSCC which found that the most frequent mutations were in *TP53* (72 %), *FAT1* (23 %), *CDKN2A* (22 %), *PIK3CA* (21 %)*, NOTCH1* (19 %), *KMT2D* (18 %), *NSD1* (10 %)*, CASP8* (9 %) [18], [19], [20], [21], [22], [23], [24], [25]. *CASP8* mutations were more common in our study and *DNAH7* mutations have not previously been reported to be frequently mutated gene in HNSCC.

Tumor-derived somatic mutations were detected in the saliva for 82 % of OSCC patients regardless of the primary site of the tumor or tumor stage, with VAFs ranging from 0.004 to 0.061. Mutations were not detected in the saliva of two patients. One of these patients had T2 tumor and the other had T4, so this did not appear to be related to the size of the primary tumor. However, in both patients, the VAF in their primary tumors was < 20 %, which may have reduced the mutation load in saliva beyond the level of detection with our assay.

The level of detection found in our study is similar to published studies on cfDNA levels in plasma at the time of diagnosis [26]. However, the level of cfDNA declines significantly after treatment [27]. Cui *et al*. showed that cfDNA level and mean allele frequency of mutations were much lower at 1 month than 3 months after treatment for the patients who recurred but beyond detection for those who did not recur [7]. Therefore, to detect minimal residual disease after treatment, more sensitive methods are required. Droplet digital PCR (ddPCR) is a sensitive technique for the detection of mutations in liquid biopsy samples [28]. However, ddPCR is time-consuming with PCR primers needing to be designed for individual-specific mutations in order to be detected in the saliva. Furthermore, the panel sequencing approach can be improved by using a larger panel of genes commonly mutated in HNSCC, deeper sequencing and improved bioinformatic analysis such as the INtegration of VAriant Reads (INVAR) pipeline that can detect 1 mutant molecule per 100,000 [29].

Other studies have explored using DNA from saliva to predict the presence of HNSCC, to monitor treatment and recurrence [7], [10], [30], [31], Table 3. An early paper from Sethi *et al*. used multiplex ligation-dependent probe amplification (MLPA) to study copy numbers of a panel of genes and found that amplification of two genes (PMAIP1 and PTPN1) could differentiate HNSCC patients from normal controls [30]. Wang *et al*. detected tumor DNA in saliva from 76 % of HNSCC patients overall and in 100 % of patients with cancer of the oral cavity using ddPCR. They also found that tumor DNA was detectable post-surgery in 3 patients who developed recurrence before a clinical detection but was not detected in 5 patients who did not relapse [10]. Similarly, a small longitudinal study of 11 patients with HNSCC oral cavity cancers detected mutations in saliva cfDNA from 10 of 11 (91 %) of patients at diagnosis [7] using Panelseq of 71 genes/hotspots mutated in HNSCC. They also found that in 5 of 6 patients with recurrence, mutations were detectable in cfDNA from saliva 3 months after surgery while from plasma they were detected after 6 months, indicating locoregional recurrence of oral cavity cancer in saliva before reaching plasma [7]. The liquid biopsies were able to detect recurrence before detection with conventional monitoring techniques.

Salivary DNA is not the only biomaterial that can predict the presence of OSCC. RNA and proteins have been also explored for this purpose. For example, Hu *et al*. identified five proteins such as CD59, M2BP, catalase, MRP14 and profilin that can differentiate between OSCC and healthy [32] and Li *et al*. developed a microarray of salivary RNA biomarkers including transcripts of DUSP1, IL-1β, IL8, HA3, S100P, SAT and OAZ1 for the same purpose [33].

In OSCC, the saliva will contain not only cell-free DNA but also DNA from normal cells and tumor cells that have exfoliated directly into the saliva. Currently, it is not clear which component (cellular DNA, cfDNA or whole DNA) is ideal for the detection of somatic mutations in saliva in OSCC. The studies described above used different methods to extract DNA from the saliva with some studies performing a centrifugation step to collect either the cellular DNA (cell pellet) or cfDNA (supernatant) before DNA extraction whereas others collect whole DNA (Table 3). In our study, whole DNA containing both cellular and cfDNA was extracted from saliva using the ORAgene kit from DNAGenotek, which contains a buffer that lyses cells and stabilizes the genomic DNA that can be stored at room temperature for several years.

## Conclusions

We were successful in this small feasibility study in detecting tumor-derived somatic mutations of OSCC in saliva from the majority of cases using our limited gene panel, suggesting that it may not be necessary to have patient-specific sequencing approaches to develop highly personalized panels. Improvement in the sensitivity of the assay may lead to a higher detection rate in saliva samples. The results provide additional data to support the emerging evidence that DNA analysis of saliva is likely to have an important role in the analysis of response to treatment and the early detection of relapse in OSCC. Early detection of recurrence has important implications for a potential increase in the success of salvage surgery for OSCC. This will be tested prospectively in a large clinical trial that has opened to recruitment, the Head and Neck Early Relapse Detection Study (HERD).

## Funding

This work was supported by a grant from the King’s Health Partners Research and Development Challenge Fund (R130597) to C. Mathew and colleagues and by the CRUK Early Detection and Diagnosis Committee Programme Award ‘Multimodality Early Detection of Head and Neck Cancer Recurrence (EDDCPGM\100001)’.

## CRediT authorship contribution statement

**Ahmed A. Ahmed:** . **Mateja Sborchia:** Data curation, Formal analysis, Writing – review & editing. **Hannah Bye:** Data curation, Formal analysis, Writing – review & editing. **Maria Roman-Escorza:** Formal analysis, Writing – review & editing. **Ariella Amar:** Data curation, Writing – review & editing. **Rhonda Henley-Smith:** Data curation, Writing – review & editing. **Edward Odell:** Writing – review & editing. **Mark McGurk:** Resources, Writing – review & editing. **Michael Simpson:** Conceptualization, Data curation, Writing – review & editing. **Tony Ng:** Conceptualization, Writing – review & editing, Funding acquisition. **Elinor J. Sawyer:** Conceptualization, Funding acquisition, Supervision, Writing – original draft, Writing – review & editing. **Christopher G. Mathew:** Conceptualization, Funding acquisition, Supervision, Writing – original draft, Writing – review & editing.

## Declaration of competing interest

The authors declare that they have no known competing financial interests or personal relationships that could have appeared to influence the work reported in this paper.
